# Protein degradation impairment and synapse elimination by microglia in BSN P3866A knock‐in mouse model of tauopathy

**DOI:** 10.1002/alz.090096

**Published:** 2025-01-03

**Authors:** Henika Patel, Pablo Martinez, Daniella Lopes, Nur Jury, Katie Vanderbosch, Yanwen You, Naomi Kouri, Darren M. Rothberg, Hiroaki Yaguchi, Shinya Tanaka, Koichi Wakabayashi, Ichiro Yabe, Melissa E. Murray, Cristian A Lasagna Reeves

**Affiliations:** ^1^ Department of Anatomy, Cell Biology, and Physiology, Indiana University School of Medicine, Indianapolis, IN USA; ^2^ Stark Neurosciences Research Institute, Indiana University School of Medicine, Indianapolis, IN USA; ^3^ Department of Neuroscience, Mayo Clinic, Jacksonville, FL USA; ^4^ Hokkaido University, Sapporo Japan; ^5^ Hirosaki University, Hirosaki Japan; ^6^ Mayo Clinic, Jacksonville, FL USA; ^7^ Center for Computational Biology and Bioinformatics, Indiana University School of Medicine, Indianapolis, IN USA

## Abstract

**Background:**

Tau aggregation is the major cause of several neurodegenerative tauopathies. Tau interaction with other proteins affects the formation of tau aggregates with seeding activity but less is known about its effects on tau‐seed properties. Our previous study revealed that Bassoon (BSN), a presynaptic protein, interacts with tau‐seed, exacerbating its toxicity in vivo. *Bsn*downregulation reduced tau spreading and overall pathology. Intriguingly, a parallel study associated missense mutations in *BSN* with tau aggregation in patients, prompting an investigation into the influence of genetic mutations in *BSN* on tau pathology for potential therapeutic insights.

**Method:**

We generated a knock‐in mouse model (BSNKI) harboring the disease‐associated p.Pro3866Ala mutation in endogenous *Bsn*. Cognitive and motor abilities were assessed in aged heterozygous and homozygous BSNKI mice, followed by analyses of BSN and tau patterns, gliosis, and gene expression changes in their brains. Additionally, we validated our findings in a human *BSN* mutation carrier.

**Result:**

At 10 months, BSNKI mice displayed motor impairments on the rotarod, and grip strength assays compared to WT mice. Their brains displayed somatic BSN and pathological tau accumulation, with the gene expression changes indicating alterations in microglia activation, protein degradation pathways, complement activation, and synapse pruning. We also observed an accumulation of pathological tau at the synapses and synapse engulfment by microglia. Histopathological analyses revealed robust microglia activation and co‐deposition of proteasomal subunits with BSN. The human *BSN* mutation carrier displayed inclusions of BSN and tau pathology similar to observations in the BSNKI model.

**Conclusion:**

Our BSNKI mouse model, reflecting a disease‐associated BSN mutation, revealed motor impairments and pathological tau and BSN deposits, mirroring observations in BSN mutation carriers. Notably, BSN seems to play a dual role, promoting tau aggregation and sequestering protein degradation molecules, leading to tau and protein accumulation at the soma and synapse, triggering microgliosis and neuroinflammation. These findings propose BSN as a promising therapeutic target for tauopathies, underscoring the need for further exploration to elucidate underlying mechanisms and therapeutic implications.

